# Deep learning from HE slides predicts the clinical benefit from adjuvant chemotherapy in hormone receptor-positive breast cancer patients

**DOI:** 10.1038/s41598-021-96855-x

**Published:** 2021-08-30

**Authors:** Soo Youn Cho, Jeong Hoon Lee, Jai Min Ryu, Jeong Eon Lee, Eun Yoon Cho, Chang Ho Ahn, Kyunghyun Paeng, Inwan Yoo, Chan-Young Ock, Sang Yong Song

**Affiliations:** 1 Department of Pathology and Translational Genomics, Samsung Medical Center, Sungkyunkwan University School of Medicine, 81 Irwon-ro, Gangnam-gu, Seoul, 06351 Republic of Korea; 2 Lunit Inc., Seoul, Republic of Korea; 3 Division of Breast Surgery, Department of Surgery, Samsung Medical Center, Sungkyunkwan University School of Medicine, Seoul, Republic of Korea; 4 Medical Ai Research Center, Research Institute of Future Medicine, Samsung Medical Center, Seoul, Republic of Korea

**Keywords:** Biomarkers, Translational research, Machine learning

## Abstract

We hypothesized that a deep-learning algorithm using HE images might be capable of predicting the benefits of adjuvant chemotherapy in cancer patients. HE slides were retrospectively collected from 1343 de-identified breast cancer patients at the Samsung Medical Center and used to develop the Lunit SCOPE algorithm. Lunit SCOPE was trained to predict the recurrence using the 21-gene assay (Oncotype DX) and histological parameters. The risk prediction model predicted the Oncotype DX score > 25 and the recurrence survival of the prognosis validation cohort and TCGA cohorts. The most important predictive variable was the mitotic cells in the cancer epithelium. Of the 363 patients who did not receive adjuvant therapy, 104 predicted high risk had a significantly lower survival rate. The top-300 genes highly correlated with the predicted risk were enriched for cell cycle, nuclear division, and cell division. From the Oncotype DX genes, the predicted risk was positively correlated with proliferation-associated genes and negatively correlated with prognostic genes from the estrogen category. An integrative analysis using Lunit SCOPE predicted the risk of cancer recurrence and the early-stage hormone receptor-positive breast cancer patients who would benefit from adjuvant chemotherapy.

## Introduction

Breast cancer is the most common cancer in women worldwide, and hormone-receptor (HR)-positive, lymph node-negative diseases account for nearly half of all breast cancer cases^1,2^. As excellent prognosis in many of these patients have been known, many efforts to identify those patients with high risk of recurrence, who would benefit from adjuvant chemotherapy (ACTx), were made using gene expression profiling^3–6^. Currently, several multigene assays, such as the 21-gene assay (Oncotype DX), PAM50, and Mammaprint, are used to stratify patients and guide ACTx according to the recurrence risk in HR-positive, and lymph node- negative breast cancer after extensive clinical validation^7,8^.

Despite the proven clinical utility of RS for the 21-gene assay, its effectiveness in patients with HR-positive, lymph node-negative, early stage breast cancer remains controversial, along with its financial burden in countries outside of the US^9,10^. Moreover, the instability of RNA extracted from formalin-fixed paraffin-embedded (FFPE) tissue in real-world practice might compromise its accuracy and interfere with the appropriate translation of the RS results^11^. Therefore, the development of a simpler and more efficient method for assessing recurrence risk using permanent tissue is necessary. As the RS from the 21-gene assay is mainly characterized by the proliferation genes group score (*MKI67*, *STK15*, *BIRC5, CCNB1*, and *MYBL2)* and the mitotic count is associated with the RS^7^, a comprehensive pathological examination of mitosis and other cell–cell interactions features, consistently reflects the RS.

Thus, we developed a deep learning (DL)-based HE image analyzer called Lunit SCOPE that identifies and quantifies various histological parameters from HE-stained whole slide images (WSIs). Previously, the Lunit SCOPE was shown to accurately detect tumor cells as well as other cells in a microenvironment, and it clearly predicted mitosis in each cell in breast cancer^12^. Based on The Cancer Genome Atlas (TCGA) pan-cancer analysis, Lunit SCOPE was able to predict an abundance of cancer-associated stroma in pancreatic adenocarcinoma and a consensus of molecular subtype 4 of colon cancer^13^, as well as tumor-infiltrating lymphocytes in immunogenic tumors such as renal cell carcinoma, melanoma, and urothelial cancer^14^.

As Lunit SCOPE accurately identifies the comprehensive features of HE slides, especially regarding mitotic count and the infiltration of immune cells or stromal cells, we hypothesized that histological parameters analyzed using Lunit SCOPE would predict the RS from the 21-gene assay, revealing potential prognostic and predictive biomarkers of ACTx in early stage hormone receptor-positive breast cancer.

## Results

### Detection of various cell types in the breast cancer HE slides

**Figure 1:**
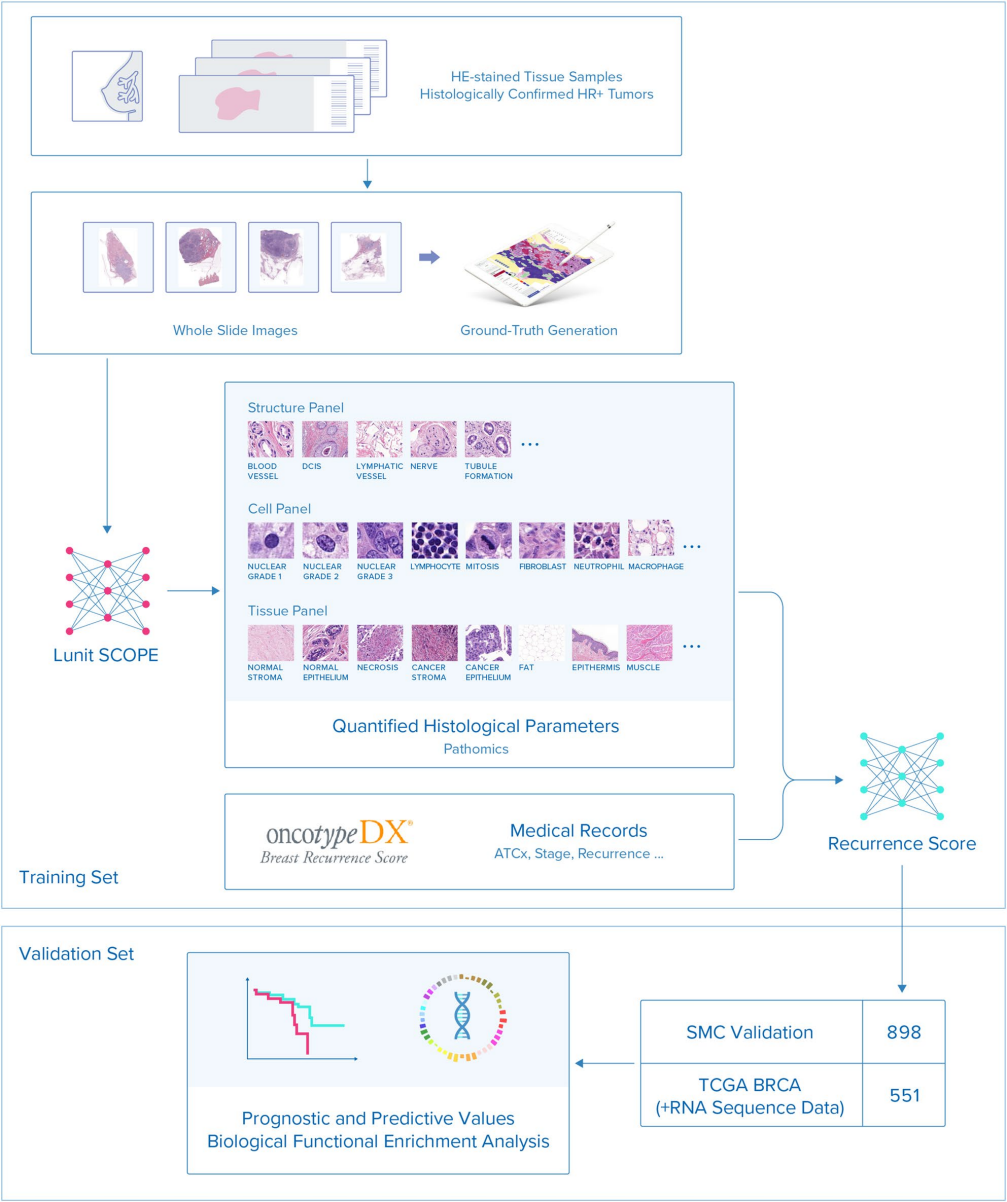
Schematic representation of Lunit SCOPE development and the workflow scheme of this study.

The Lunit SCOPE divides the HE slide image into histological parameters through three panels, including the tissue, structure, and cell panel. The process used to develop the Lunit SCOPE and workflow of this study are illustrated in Fig. 1 (detailed description in the Supplementary Methods). Each panel is an independent multi-class prediction model trained using curated ground-truth annotations from expert pathologists. The panels decipher the histological parameters in the image divided into small patch images and ultimately return the aggregated count values corresponding to the tissue, structure, and cell from the WSIs. The performance of the three panels is described in Supplementary Table 1.

### Development of a model to predict risk group based on histological parameters

**Figure 2:**
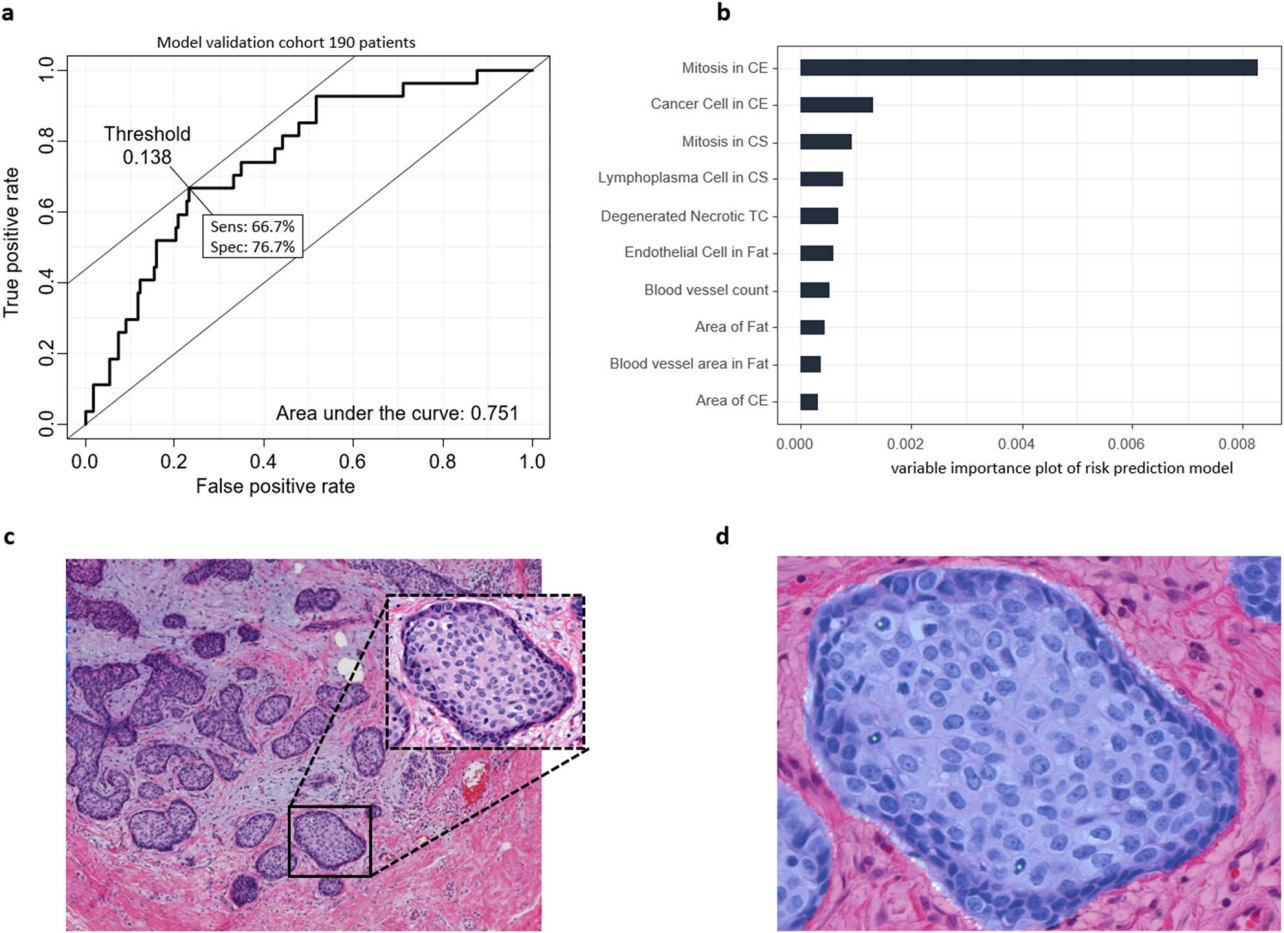
ROC curve for validation set and relative feature importance with example patch. (**a**) The receiver operating characteristic (ROC) curve on 190 model validation set and decision threshold for RS > 25 positivity or negativity. (**b**) Top 10 important pathological parameters to predict the Oncotype DX score. (c) WSI patch of high-risk patients and highlighted epithelium. (**d**) Segmented regions for cancer epithelium and mitotic cells detected by Lunit SCOPE (cyan).

The study included a total of 1875 patients with HE-stained WSIs and clinical information, including cancer recurrence and survival (Table 1). Of the 445 patients with a 21-gene assay score provided by Oncotype DX, 255 images with long-term follow-up clinical information were used as a training dataset to predict the RS using histologic parameters derived by Lunit SCOPE. The remaining 190 images were used to estimate the predictive performance of the model. The validity of the trained risk prediction in model validation cohort was 0.751 for the area under the receiver operating characteristics curve (AUROC) (Fig. 2a). The optimal classification threshold is defined as the cut point with the maximum sensitivity + specificity.

**Table 1:** Clinical characteristics of the hormone receptor-positive breast cancer patients for the model development cohort, the prognosis validation cohort, and TCGA BRCA cohort.

Variables	Model development	Prognosis validation	TCGA BRCA
No	255	898	532
Age	45.89 (7.83)	53.34 (1.92)	59.92 (13.28)
**Sex**			
Female	255	898	525
Male	0	0	7
**Stage**			
I	248	636	100
II	3	246	305
III	0	16	127
IV	0	0	0
**Subtype**			
ER+	255	889	522
PR+	245	843	455
HER2+	0	0	0
**AdjCTx**			
Yes	33	535	–
No	222	363	–
**AdjHTx**			
Yes	–	868	–
No	–	30	–
**Menopausal**			
Pre	204	602	362
Post	51	287	138
Follow-up years	2.28 (1.96–4.10)	9.13 (8.17–9.92)	1.92 (0.46–3.01)
**Oncotype DX**			
> 25	21		
≤ 25	234		

*AdjCTx* adjuvant chemotherapy, *AdjHTx* hormone therapy.

The top 10 important histological parameters for predicting the RS > 25 based on the 21-gene assays are listed in the variable importance plot (Fig. 2b). The most important variable for predicting the RS of the 21-gene assay was the mitotic cell count located in the cancer epithelium, followed by cancer cell. Top 4 important variables were in the cancer epithelium (CE) and cancer stroma (CS) domains. The other histologic parameters that were not included in the list represented low counted values, which were filtered out in the histologic parameter preprocessing step. Examples of cancer epithelium regions and mitotic cells highlighted in high-risk patients are shown in Fig. 2c,d.

### Clinical validation of prediction model in an independent cohort

**Figure 3:**
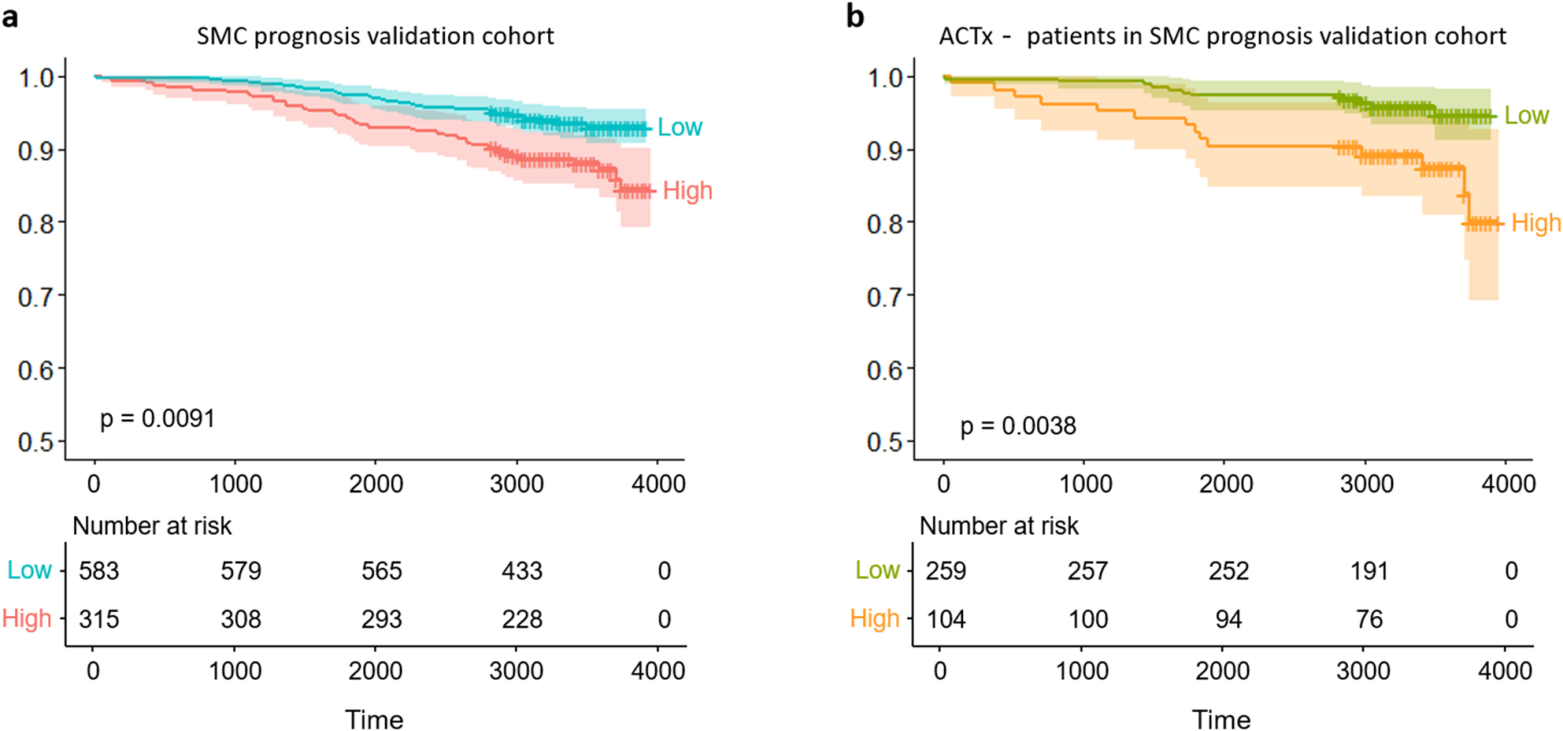
Time to disease recurrence survival analysis using the prognosis validation cohort. (**a**) The overall patient DFS was divided into two groups based on the predicted Oncotype DX threshold score in the prognosis validation cohort. (**b**) DFS of patients without adjuvant chemotherapy treatment.

The RS values of the 898 SMC prognosis validation cohort and 532 TCGA cohort were used to validate the Lunit SCOPE model. The mean value for the output of the SMC model development cohort and validation cohort were 0.040 and 0.090, respectively (Supplementary Figure 1). The time to disease recurrence and survival analysis by risk group (threshold = 0.138) was performed in both cohorts. Patients in the high-risk group had significantly poorer survival than those in the low-risk group (*p* < 0.01) (Fig. 3a). In the multivariate Cox proportional hazard model, which included clinical variables, the predicted risk was most significant (*p* < 0.01), with a 3.128 coefficient followed by the T-stage, N-stage, age, and adjuvant chemotherapy. The details of the multivariate and univariate Cox proportional hazard models for disease-free survival (DFS) in the prognosis validation cohort are shown in Supplementary Table 2.

To confirm the utility of our model, DFS of each risk group was compared according to whether ACTx was done or not. From the 363 patients who did not receive ACTx, the 104 high-risk patients had a lower survival rate than the low-risk patients (*p* < 0.01) (Fig. 3b). However, for the 535 patients who received ACTx, there was no difference between the prognosis of the two risk groups according to the predicted risk (*p* = 0.120) and multivariate analysis with age, T-stage, and N-stage (*p* = 0.117) (Supplementary Figure 2). Further, we divided all patients into four groups according to their ACTx status and a predicted risk. The log-rank p-value for the survival analysis of the four groups showed a significantly (*p* < 0.01) worse prognosis in high-risk patients without ACTx. ACTx status in 583 low-risk predicted patients was no significant difference in cancer recurrence and survival (*p* = 0.092). The clinical characteristics of the four groups divided by the predicted risk and adjuvant treatment are summarized in Supplementary Table 3.

532 TCGA breast cancer cohort was used as the external validation set. The survival rate of TCGA cohort was worse than that of the prognosis validation cohort (*p* < 0.001), while the median output of the former cohort was higher than that of the latter. Based on Lunit SCOPE predictions, among the 532 HR- positive breast cancer, high risk group showed significantly worse prognoses in cox proportional hazard model (*p* = 0.023) with the more advanced stages of cancer (Fisher’s exact test, *p* = 0.024).

Predicted risk increased significantly with increasing stage, in both the prognosis validation cohort and the TCGA cohort (*p* < 0.001). Age was not significantly correlated in both cohorts using Kendal's method, but age was a variable that was not significant in survival in both cohorts. The distribution of predicted risk by cancer stage and age was shown in Supplementary Figure 3.

### Distinct genomic and transcriptomic characteristics of the predicted risk in TCGA

**Figure 4:**
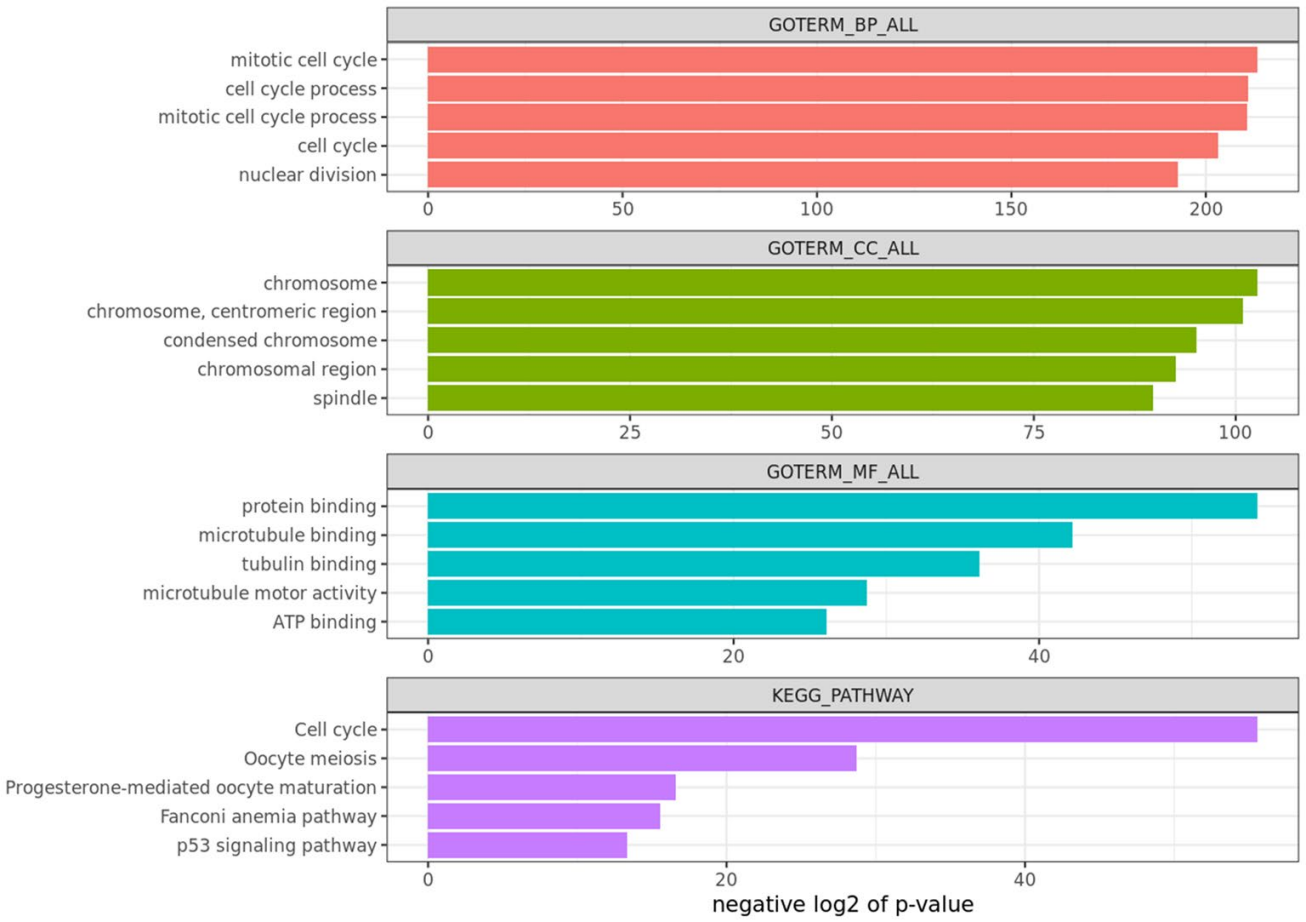
Functional enrichment analysis of the top 300 correlated genes with the predicted risk in TCGA BRCA cohort.

We analyzed TCGA cohort gene expression data associated with the predicted risk using 532 diagnostic slide images. The top 300 genes that had the highest correlation coefficient with the predicted risk were used for the functional enrichment analysis of the BP, CC, and MF for the Gene Ontology and Kyoto Encyclopedia of Genes and Genomes (KEGG) pathways. Based on the Bonferroni-corrected significance threshold (*p* < 0.05), 228 significant Gene Ontology and KEGG pathway terms were identified. The top-5 gene ontology functional terms and pathways are shown in Fig. 4, with negative log2 based *p*-values. Mitotic cell cycle, cell cycle process, cell cycle, nuclear division, and cell division were the enriched biological processes observed following Gene Ontology analysis of the top 300 genes. Among the various cellular parameters, spindle and chromosome, which play an important role in the cell cycle, were significantly enriched. Furthermore, protein binding was significantly enriched. The cell cycle was identified as another significant term in the KEGG analysis. The details of the functional terms, genes, and significance of the top 100 functions are available in Supplementary Table 4.

Of the 21 genes assessed during the Oncotype DX test, the correlations of 16 genes with the predicted risk, excluding the reference gene, were measured and ordered by correlation coefficient (Table 2). The genes from the proliferation category, including AURKA, MYBL2, MKI67, BIRC5, and CCNB1, were positively correlated with the predicted risk, while the estrogen receptor genes, including ESR1, PGR, SCUBE2, and BCL2, were negatively correlated or not significantly. The other genes, including invasion-associated genes and HER2, had significantly lower correlations than those in the proliferation and estrogen receptor categories (Wilcoxon rank sum test, *p* = 0.003, *p* = 0.006).

**Table 2:** Correlation between the predicted risk and the genes from the Oncotype DX gene assay.

Genes	Cor	*p*-value	Category
AURKA	0.380	1.2.E−19	Proliferation
MYBL2	0.341	1.3.E−15	Proliferation
MKI67	0.335	4.2.E−15	Proliferation
BIRC5	0.332	7.6.E−15	Proliferation
CCNB1	0.312	3.9.E−13	Proliferation
CD68	0.123	4.7.E−03	Other genes
CTSL2	0.123	4.8.E−03	Invasion
MMP11	0.048	2.7.E−01	Invasion
ESR1	0.041	3.4.E−01	Estrogen
GRB7	0.000	9.9.E−01	HER2
GSTM1	− 0.024	5.9.E−01	Other genes
ERBB2	− 0.032	4.7.E−01	HER2
PGR	− 0.094	3.1.E−02	Estrogen
BCL2	− 0.098	2.5.E−02	Estrogen
BAG1	− 0.109	1.3.E−02	Other genes
SCUBE2	− 0.129	3.1.E−03	Estrogen

## Discussion

We developed a DL-based HE image analyzer called Lunit SCOPE to identify and quantify various histological parameters from HE-stained WSIs. Using the pathological features derived from Lunit SCOPE, we developed a prediction model for the 21-gene assay RS obtained using Oncotype DX; thus, revealing potential for prognostic and predictive biomarkers of ACTx for early stage HR-positive breast cancers patients. high-risk predicted patients had significantly worse prognoses than the low risk patients (Fig. 3b). In addition to these prognostic capabilities, our findings might have a significant clinical impact on the financial burden of early stage breast cancer. Moreover, gene set enrichment analysis showed that the predicted risk was associated with pathways involved in the cell cycle and nuclear division, which are associated with a high risk of recurrence.

Recent advances in DL analysis have shed light on novel approaches for understanding cancer biology. Growing evidence shows that DL analyses of medical images are clinically reliable tools for diagnosis^15–17^. However, the clinical significance of this technology as a predictive biomarker has not yet been reported. Lunit SCOPE was developed using > 1000 annotated breast cancer slides containing various cell types and tissue architectures. The preliminary results showed that Lunit SCOPE accurately predicted tumor proliferation in breast cancer, and provided a core biological explanation as to how the 21-gene expression assay works in predicting high-risk patients through the evaluation of proliferation genes^12^. Moreover, Lunit SCOPE detected cancer-associated fibroblasts that disrupt the stromal barrier and induce the infiltration of tumor-associated macrophages^18,19^, which is indicative of cancer aggressiveness. Therefore, we hypothesized that Lunit SCOPE could predict high-risk patients who would benefit from ACTx.

The 21-gene expression assay test included proliferation, estrogen, HER2, invasion, and other cancer-related gene categories. Based on the Lunit SCOPE predictions using pathology images, the five genes associated with cancer proliferation had a positive correlation with the predicted risk. This suggests that the expression of proliferation, cell cycle, and progression genes ultimately affected the components of the pathology image, which were associated with cancer recurrence. Excluding ESR1, which was not significant, three genes in the estrogen category were negatively correlated with the predicted risk. The *PGR* (progesterone receptor), *BCL2* Apoptosis Regulator and *SCUBE2* (Signal Peptide, CUB Domain And EGF Like Domain Containing 2) are known to be a favorable prognostic marker on breast cancer recurrence^20–22^. The directionality of the correlations between the expression of recurrence-related genes and the predicted risk indicates that the pathology-based predictions of this model were consistent with those obtained using the 21-gene expression assay.

There are several limitations to the current study. First, the RS of the model development cohort did not have a range that was sufficient to predict RS. Recent clinical trials have shown that endocrine treatment alone is not inferior to endocrine treatment plus chemotherapy in patients with an RS of 11–25, and a more well-validated RS cutoff for the decision to add chemotherapy to the standard treatment would be 25^8^. The cutoff of 21 gene-assay changes based on age 50, but this model predicted based on pathology image does not reflect age. Therefore, this model can underestimate the risk of young patients. Another limitation was represented by the selection bias present in the retrospective analysis, as patients who did not receive chemotherapy were associated with other clinical factors, such as poor performance status or poor compliance. Moreover, physicians would choose patients who are clinically high-risk to receive ACTx. This factor could contribute to worse clinical outcomes in patients with ACTx compared to those without ACTx. To overcome this limitation, a well-designed prospective clinical trial is required.

In conclusion, the Lunit SCOPE predicted the early stage HR-positive breast cancer patients with a high risk of recurrence, as well as those who would benefit from adjuvant chemotherapy.

## Methods

### Patients and tumor tissues for pathology slides

The protocol for this retrospective study was approved by the Ethics Committee of the Institutional Review Board (IRB 2018-03-038-002) of Samsung Medical Center (SMC). Informed consent was also waived by Ethics Committee of the Institutional Review Board. All experiments were performed in accordance with relevant guidelines and regulations and all experimental protocols were approved by SMC. A total of 1343 pathology slide images, derived from anonymized HE-stained tissue samples from breast cancer patients with histologically confirmed hormone receptor-positive tumors, were acquired using a WSI scanner (Pannoramic 1000, 3DHISTECH Ltd., Budapest, Hungary) at a magnification of 40 ×. Of the total of 445 images from patients with a 21-gene assay RS obtained from Oncotype DX (Genomic Health, Redwood City, CA, USA), 255 images with clinical information were used to develop the model predicting the high risk of recurrence (RS > 25), and the 190 images with RS were used as a validation cohort to estimate the predictive performance using AUROC. We have used the HE images from the same block that were used for Oncotype DX test to minimize possible problems due to intratumoral heterogeneity^23^. The remaining 898 images without RS were used as a prognosis validation cohort to confirm the prognostic and predictive values of the predicted risk.

A total of 532 samples with both digital pathology images and image-matched RNA sequencing data from primary tumor tissues from the TCGA BRCA cohort were also included in the data analysis. Data from the HR-positive and human epidermal growth factor receptor-2 (HER2) negative cases (excluding advanced stage patients) were used for the external validation of the prognostic significance assessment^24^.

### Development of the DL model

For training, anonymized HE-stained tissue slides were reviewed by expert pathologists (SYC, EYC, and SYS). The informative regions from these slides were manually selected and annotated by expert pathologists. Next, we trained convolutional neural networks (CNNs) to decipher various types of histologic parameters^25^. The WSIs were tiled into 50% overlapping 4096 × 4096 patches to analyze and quantify the histologic parameters. The performance of these models was evaluated by measuring the distance between the outputs of two images using the validation set with accuracy, intersection over union (IoU), and mean average precision (mAP).

### Raw count of histological parameter preprocessing

The histological parameters that were quantified using Lunit SCOPE had a count distribution based on tissue, structure, and cell type. We applied the Trimmed Mean of M-values (TMM) count normalization for the histological parameters count to make accurate data proportions comparisons between samples without missing the data composition^26^.

### TCGA RNA sequencing data analyses

RNA-seq data for breast cancers were obtained from TCGA Broad Institute GDAC Firehose. The RNA sequencing raw count samples, quantified using RNA-seq expectation maximization^27^. To filter out the genes with low expression levels, the genes with counts per million (cpm) values < 1 in at least half of the samples were excluded^28^. The raw read counts were normalized using TMM and logCPM transformation with limma voom. Finally, the expression levels of 17,649 genes were used for this analysis^29^.

To determine the biological functions associated with the predicted risk based on the 21-gene assay, we performed a Pearson correlation analysis. The top 300 highly correlated genes were selected as related genes, and an enrichment analysis was performed for the BP, CC, and MF terms in the Gene Ontology and KEGG pathway database using the RDAVIDWebService tool in Bioconductor^30–32^.

### Prediction of RS using random forest (RF) regression

Fast unified RFs for survival, regression, and classification (RF-SRC), a non-parametric statistical estimation was used to predict the RS from the 21-gene assay based on Lunit SCOPE^33^. The RF model was trained with the out-of-bag (OOB) training data from 255 images with binarized 21-gene assay (RS > 25). The method provides the importance index of the input variable for classification with the reprioritization component of RS assessments. The model was developed using bootstrap samples with RS, and the OOB samples were used as test samples. A variable’s importance was defined as the mean decrease in the tree’s performance for the randomly permuted OOB samples. The loss of function for minimizing the gini was used for the model assessment metrics in the classification problem to assess the goodness-of-fit and predictive performance of the RS from the 21-gene assay.

## Supplementary Information

Supplementary Information 1.

Supplementary Information 2.
