# Author Correction: Deep learning from HE slides predicts the clinical benefit from adjuvant chemotherapy in hormone receptor-positive breast cancer patients

**DOI:** 10.1038/s41598-021-00546-6

**Published:** 2021-10-20

**Authors:** Soo Youn Cho, Jeong Hoon Lee, Jai Min Ryu, Jeong Eon Lee, Eun Yoon Cho, Chang Ho Ahn, Kyunghyun Paeng, Inwan Yoo, Chan-Young Ock, Sang Yong Song

**Affiliations:** 1 Department of Pathology and Translational Genomics, Samsung Medical Center, Sungkyunkwan University School of Medicine, 81 Irwon-ro, Gangnam-gu, Seoul, 06351 Republic of Korea; 2 Lunit Inc., Seoul, Republic of Korea; 3 Division of Breast Surgery, Department of Surgery, Samsung Medical Center, Sungkyunkwan University School of Medicine, Seoul, Republic of Korea; 4 Medical Ai Research Center, Research Institute of Future Medicine, Samsung Medical Center, Seoul, Republic of Korea

Correction to: *Scientific Reports* 10.1038/s41598-021-96855-x, published online 30 August 2021

In the original version of this Article, Sang Yong Song was omitted as a corresponding author. Correspondence and requests for materials should also be addressed to yodasong@gmail.com.

The original Article and accompanying Supplementary Information file have been corrected.

