# Supplementary material for: Deep learning from HE slides predicts the clinical benefit from adjuvant chemotherapy in hormone receptor-positive breast cancer patients
Source: Sci Rep. 2021 Aug 30;11:17363. doi: 10.1038/s41598-021-96855-x (PMC8405682; doi:10.1038/s41598-021-96855-x)
Supplement: Supplementary file 1 — Supplementary Information 1. [file 41598_2021_96855_MOESM1_ESM.docx]

**Supplementary Figure 1**. DFS and distribution of the predicted RS in three groups, SMC model development cohort, SMC prognosis validation cohort and TCGA BRCA external validation cohort. a) Kaplan-Meier plot for DFS. b) The median predicted RS for the four cohorts were 0.085, 0.111, 0.124 and 0.307 respectively.


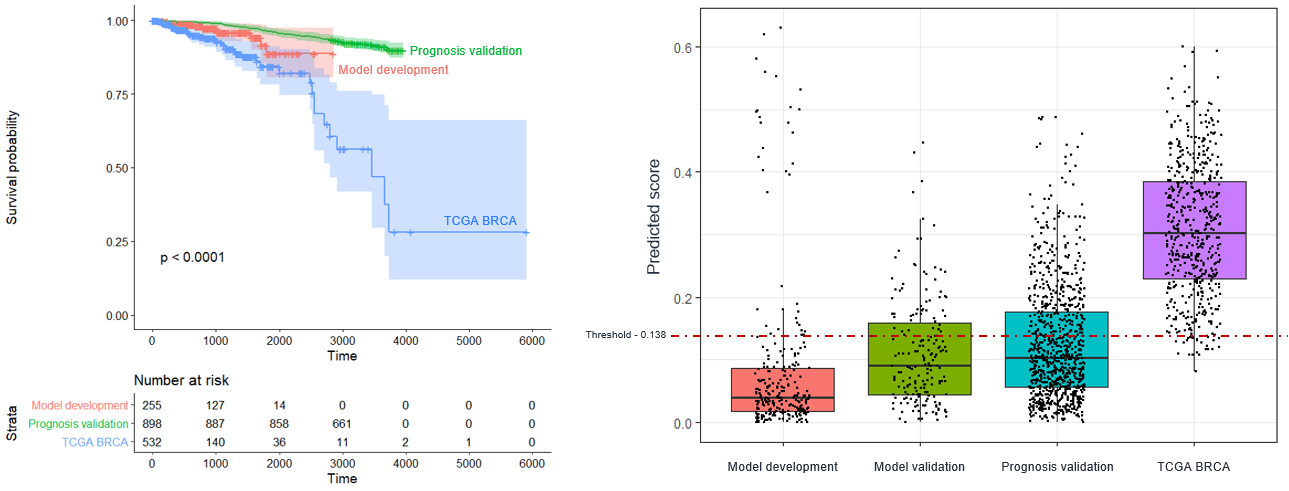


**Supplementary Figure 2**. Survival analysis of the SMC prognosis validation cohort in detail. a) DFS analysis for the high/low risk patients in the prognosis validation cohort with ACTx. b) DFS analysis for the low-risk predicted SMC validation cohort with or without ACTx.


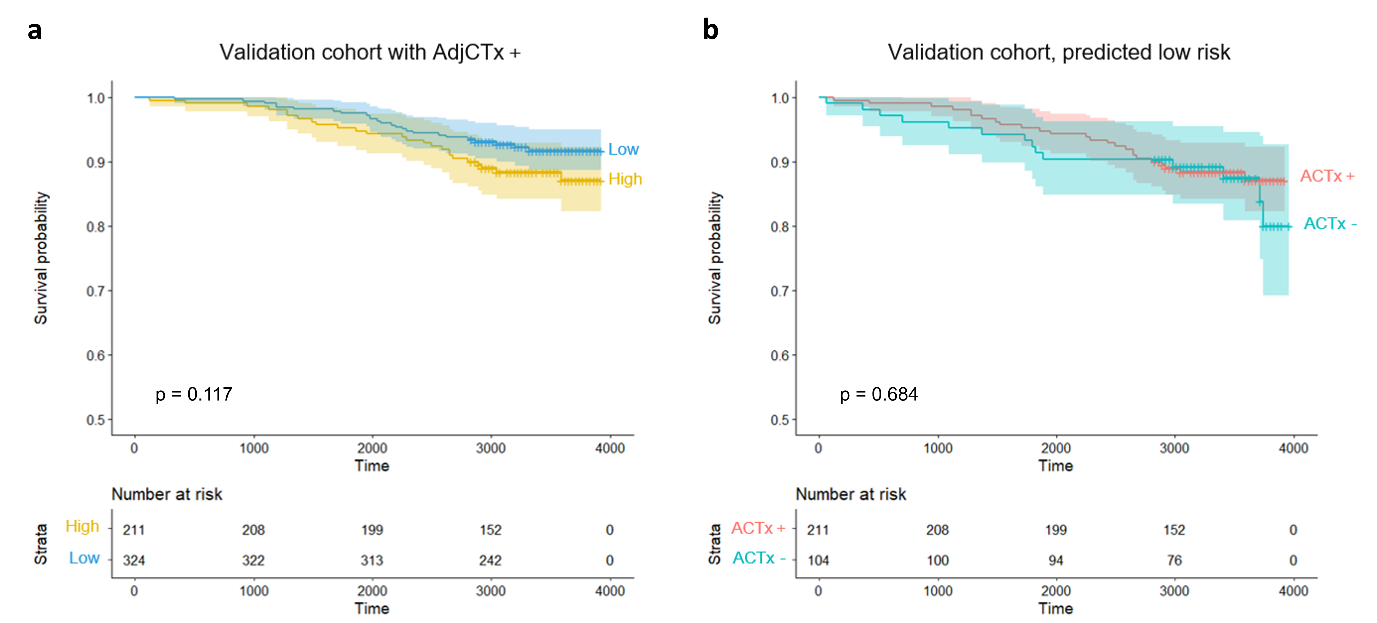


**Supplementary Figure 3**. The distribution of predicted RS by cancer stage (a) and age (b).


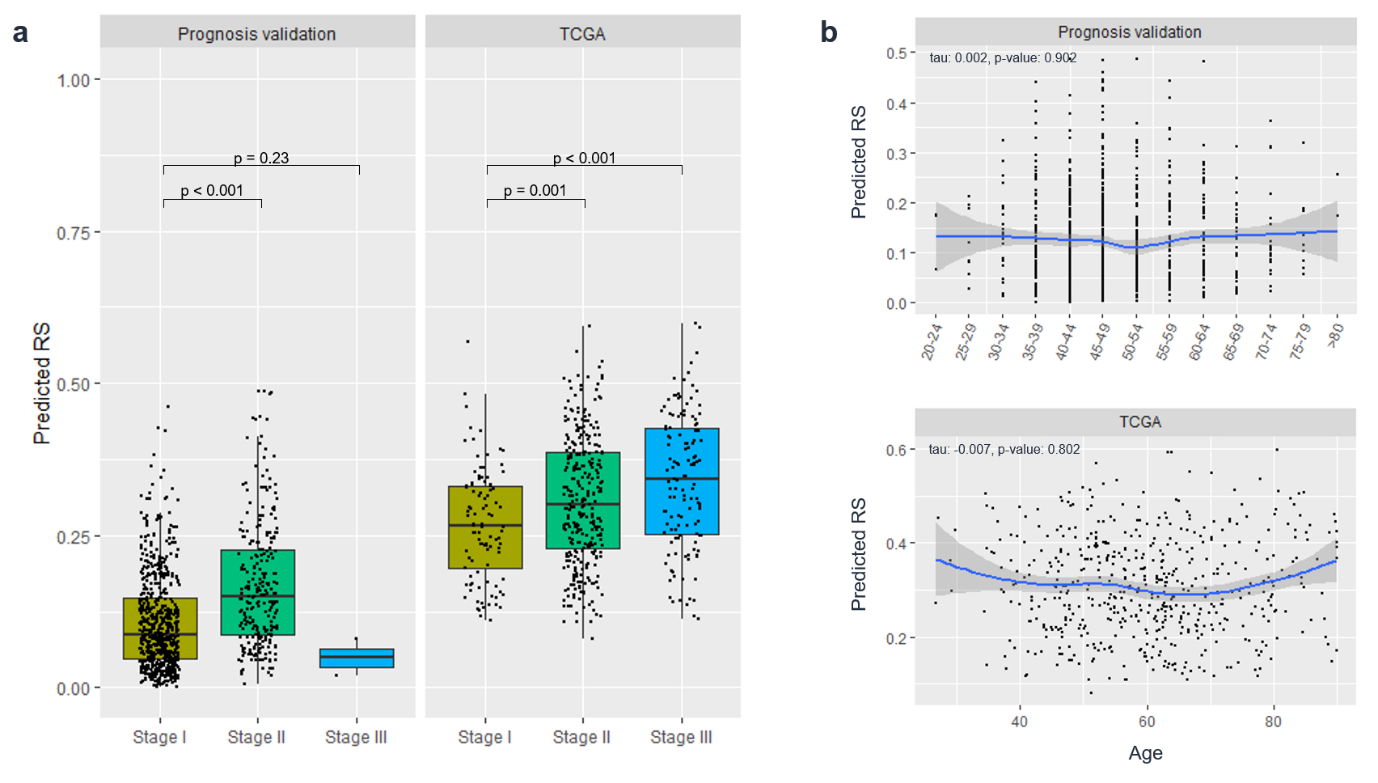


**Supplementary Table 1**. Performance of Lunit-SCOPE according to three different tasks on tissue, structure, and cells.

| **Tissue (Acc)** | | | | | | | | | | |
| --- | --- | --- | --- | --- | --- | --- | --- | --- | --- | --- |
| **CE** | | **CS** | **NR** | | **Nec** | | **Fat** | | **BG** | |
| **0.9135** | | **0.7629** | **0.9271** | | **0.8784** | | **0.9595** | | **0.8300** | |
| **Structure (IoU)** | | | | | | | | | | |
| **Background** | | **TF** | **DCIS** | | **N** | | **BV** | | **LV** | |
| **0.9460** | | **0.6589** | **0.9778** | | **0.9290** | | **0.8907** | | **0.6194** | |
| **Cell (AP)** | | | | | | | | | | |
| **LC** | **FB** | **MA** | **TC1** | **TC2** | | **TC3** | **DC** | **EC** | | **MT** |
| **0.8773** | **0.6063** | **0.2987** | **0.2921** | **0.5992** | | **0.6340** | **0.5640** | **0.4097** | | **0.7863** |

**Supplementary Table 2**. Univariate and multivariate analyses of DFS in the SMC validation cohort (n=898).

| **Univariate analysis** | | | | | |
| --- | --- | --- | --- | --- | --- |
|  | **coef** | **HR** | **z** | **P.value** |  |
| Predicted risk | 3.347 | 28.419 (9.757, 82.765) | 3.131 | 0.002 | ** |
| AdjCTx | 0.297 | 1.346 (0.833, 2.176) | 1.213 | 0.225 |  |
| Age | 0.095 | 1.100 (0.982, 1.232) | 1.651 | 0.099 | * |
| T stage |  |  |  |  |  |
| T2 vs. T1 | 0.366 | 1.442 (0.886, 2.346) | 1.472 | 0.141 |  |
| T3 vs. T1 | 1.342 | 3.827 (1.377, 10.635) | 2.574 | 0.01 | ** |
| N stage |  |  |  |  |  |
| N1 vs. N0 | 0.581 | 1.788 (1.015, 3.149) | 2.012 | 0.044 | . |
| **Multivariate analysis** | | | | | |
|  | **coef** | **HR** | **z** | **P.value** |  |
| Predicted risk | 3.128 | 22.838 (7.338, 71.086) | 2.755 | 0.006 | ** |
| AdjCTx | 0.143 | 1.154 (0.868, 1.535) | 0.503 | 0.615 |  |
| Age | 0.109 | 1.115 (1.050, 1.184) | 1.816 | 0.069 | . |
| T stage |  |  |  |  |  |
| T2 vs. T1 | 0.097 | 1.115 (0.839, 1.446) | 0.356 | 0.722 |  |
| T3 vs. T1 | 1.24 | 3.444 (2.032, 5.837) | 2.344 | 0.019 | * |
| N stage |  |  |  |  |  |
| N1 vs.N0 | 0.555 | 1.742 (0.305, 9.941) | 1.83 | 0.067 | . |

AdjCTx: Adjuvant Chemotherapy, HR: Hazard ratio

**Supplementary Table 3**. Clinical characteristics of the four groups of validation cohort divided on the basis of the predicted RS and adjuvant treatment.

|  | Low-risk AdjCTx- | Low-risk AdjCTx+ | High-risk AdjCTx- | High-risk AdjCTx+ |
| --- | --- | --- | --- | --- |
| N | 259 | 324 | 104 | 211 |
| age | 58.84 (21.30) | 49.01 (15.00) | 59.71 (23.83) | 50.09 (17.43) |
| T-stage 1 2 3 4 | 246  13  0  0 | 217  98  9  0 | 78  24  2  0 | 95  111  5  0 |
| n-stage 0 1 | 253  6 | 255  69 | 99  5 | 178  33 |
| Follow-up  (years) | 8.88 (7.62-10.14) | 8.9 (7.49-10.31) | 8.69 (6.58-10.8) | 8.84 (7.15-10.52) |
| Recurrence YES NO | 11  248 | 25  299 | 14  90 | 25  186 |

**Supplementary Table 4.** Significantly associated functional terms in the enrichment analysis of the top 300 genes correlated with the predicted RS.

| **Category** | **Term** | **Count** | **Fold**  **Enrichment** | **FDR** |
| --- | --- | --- | --- | --- |
| GOTERM_BP_ALL | mitotic cell cycle | 107 | 7.37 | 2.16E-59 |
| GOTERM_BP_ALL | cell cycle process | 121 | 5.94 | 4.70E-59 |
| GOTERM_BP_ALL | mitotic cell cycle process | 103 | 7.71 | 4.70E-59 |
| GOTERM_BP_ALL | cell cycle | 129 | 5.14 | 6.02E-57 |
| GOTERM_BP_ALL | nuclear division | 84 | 9.60 | 6.71E-54 |
| GOTERM_BP_ALL | organelle fission | 84 | 9.00 | 1.20E-51 |
| GOTERM_BP_ALL | chromosome segregation | 66 | 13.17 | 1.16E-49 |
| GOTERM_BP_ALL | mitotic nuclear division | 72 | 11.13 | 1.24E-49 |
| GOTERM_BP_ALL | nuclear chromosome segregation | 59 | 13.70 | 9.88E-45 |
| GOTERM_BP_ALL | sister chromatid segregation | 53 | 15.77 | 6.43E-43 |
| GOTERM_BP_ALL | cell division | 73 | 8.46 | 5.45E-42 |
| GOTERM_BP_ALL | mitotic sister chromatid segregation | 39 | 18.30 | 9.17E-33 |
| GOTERM_CC_ALL | chromosome | 72 | 5.23 | 5.08E-27 |
| GOTERM_BP_ALL | chromosome organization | 80 | 4.50 | 7.33E-27 |
| GOTERM_CC_ALL | chromosome, centromeric region | 38 | 13.67 | 8.87E-27 |
| GOTERM_BP_ALL | cell cycle phase transition | 56 | 6.82 | 9.49E-26 |
| GOTERM_BP_ALL | mitotic cell cycle phase transition | 54 | 7.00 | 3.32E-25 |
| GOTERM_BP_ALL | regulation of cell cycle | 71 | 4.85 | 4.79E-25 |
| GOTERM_CC_ALL | condensed chromosome | 38 | 12.30 | 3.33E-25 |
| GOTERM_CC_ALL | chromosomal region | 45 | 8.84 | 1.37E-24 |
| GOTERM_CC_ALL | spindle | 42 | 9.43 | 7.75E-24 |
| GOTERM_BP_ALL | regulation of cell cycle process | 55 | 6.16 | 4.65E-23 |
| GOTERM_CC_ALL | chromosomal part | 63 | 5.18 | 2.23E-23 |
| GOTERM_BP_ALL | single-organism organelle organization | 86 | 3.55 | 2.89E-22 |
| GOTERM_BP_ALL | sister chromatid cohesion | 30 | 15.62 | 5.13E-22 |
| GOTERM_CC_ALL | kinetochore | 30 | 15.45 | 2.69E-22 |
| GOTERM_BP_ALL | organelle organization | 131 | 2.33 | 1.40E-20 |
| GOTERM_CC_ALL | condensed chromosome, centromeric region | 27 | 16.39 | 2.01E-20 |
| GOTERM_BP_ALL | regulation of mitotic cell cycle | 47 | 6.38 | 1.17E-19 |
| GOTERM_BP_ALL | regulation of chromosome segregation | 24 | 19.04 | 6.59E-19 |
| GOTERM_CC_ALL | condensed chromosome kinetochore | 25 | 16.50 | 9.66E-19 |
| GOTERM_BP_ALL | regulation of mitotic nuclear division | 27 | 12.85 | 4.00E-17 |
| GOTERM_CC_ALL | microtubule cytoskeleton | 63 | 3.89 | 4.45E-17 |
| GOTERM_BP_ALL | regulation of nuclear division | 28 | 11.38 | 1.74E-16 |
| GOTERM_BP_ALL | cell cycle checkpoint | 31 | 9.02 | 9.40E-16 |
| GOTERM_CC_ALL | cytoskeletal part | 71 | 3.12 | 1.34E-14 |
| GOTERM_BP_ALL | microtubule cytoskeleton organization | 38 | 5.92 | 5.26E-14 |
| GOTERM_CC_ALL | nucleoplasm | 103 | 2.34 | 1.67E-14 |
| GOTERM_BP_ALL | regulation of mitotic cell cycle phase transition | 33 | 7.05 | 7.73E-14 |
| GOTERM_BP_ALL | regulation of cell cycle phase transition | 34 | 6.74 | 8.12E-14 |
| GOTERM_BP_ALL | cellular component organization | 158 | 1.72 | 1.76E-13 |
| KEGG_PATHWAY | Cell cycle | 23 | 11.29 | 2.64E-13 |
| GOTERM_CC_ALL | intracellular non-membrane-bounded organelle | 121 | 2.04 | 1.16E-13 |
| GOTERM_CC_ALL | non-membrane-bounded organelle | 121 | 2.04 | 1.16E-13 |
| GOTERM_MF_ALL | protein binding | 220 | 1.36 | 2.68E-12 |
| GOTERM_BP_ALL | DNA metabolic process | 55 | 3.68 | 5.86E-13 |
| GOTERM_BP_ALL | cellular component organization or biogenesis | 159 | 1.69 | 7.89E-13 |
| GOTERM_BP_ALL | positive regulation of cell cycle process | 29 | 7.70 | 9.70E-13 |
| GOTERM_BP_ALL | microtubule-based process | 43 | 4.64 | 1.17E-12 |
| GOTERM_BP_ALL | mitotic spindle organization | 15 | 24.99 | 3.25E-12 |
| GOTERM_CC_ALL | nuclear lumen | 111 | 2.10 | 1.13E-12 |
| GOTERM_BP_ALL | DNA replication | 30 | 6.89 | 5.71E-12 |
| GOTERM_BP_ALL | positive regulation of cell cycle | 32 | 6.29 | 6.15E-12 |
| GOTERM_BP_ALL | regulation of sister chromatid segregation | 17 | 16.91 | 2.13E-11 |
| GOTERM_BP_ALL | mitotic cell cycle checkpoint | 23 | 9.64 | 2.22E-11 |
| GOTERM_CC_ALL | condensed chromosome outer kinetochore | 10 | 56.67 | 1.51E-11 |
| GOTERM_BP_ALL | regulation of mitotic sister chromatid separation | 15 | 20.40 | 7.45E-11 |
| GOTERM_BP_ALL | chromosome separation | 17 | 15.52 | 8.45E-11 |
| GOTERM_BP_ALL | metaphase/anaphase transition of cell cycle | 15 | 19.99 | 9.72E-11 |
| GOTERM_BP_ALL | mitotic sister chromatid separation | 15 | 19.60 | 1.30E-10 |
| GOTERM_BP_ALL | cytokinesis | 20 | 11.11 | 1.32E-10 |
| GOTERM_BP_ALL | cytoskeleton organization | 55 | 3.20 | 1.86E-10 |
| GOTERM_BP_ALL | negative regulation of mitotic cell cycle | 25 | 7.61 | 1.99E-10 |
| GOTERM_CC_ALL | microtubule | 32 | 5.39 | 1.16E-10 |
| GOTERM_BP_ALL | regulation of mitotic sister chromatid segregation | 15 | 17.85 | 4.97E-10 |
| GOTERM_BP_ALL | meiotic cell cycle | 25 | 7.24 | 5.71E-10 |
| GOTERM_CC_ALL | midbody | 20 | 10.23 | 1.79E-10 |
| GOTERM_CC_ALL | spindle pole | 20 | 10.23 | 1.79E-10 |
| GOTERM_CC_ALL | nuclear part | 113 | 1.93 | 2.00E-10 |
| GOTERM_CC_ALL | spindle microtubule | 15 | 17.29 | 2.22E-10 |
| GOTERM_BP_ALL | regulation of mitotic metaphase/anaphase transition | 14 | 19.85 | 9.06E-10 |
| GOTERM_BP_ALL | regulation of metaphase/anaphase transition of cell cycle | 14 | 19.44 | 1.20E-09 |
| GOTERM_BP_ALL | negative regulation of cell cycle process | 25 | 6.94 | 1.38E-09 |
| GOTERM_MF_ALL | microtubule binding | 24 | 7.34 | 5.97E-09 |
| GOTERM_BP_ALL | cell cycle G2/M phase transition | 23 | 7.78 | 1.50E-09 |
| GOTERM_CC_ALL | intracellular organelle lumen | 118 | 1.85 | 5.93E-10 |
| GOTERM_CC_ALL | organelle lumen | 119 | 1.84 | 7.33E-10 |
| GOTERM_CC_ALL | cytoskeleton | 73 | 2.42 | 1.44E-09 |
| GOTERM_CC_ALL | membrane-enclosed lumen | 119 | 1.82 | 1.71E-09 |
| GOTERM_BP_ALL | DNA conformation change | 26 | 6.10 | 7.36E-09 |
| GOTERM_BP_ALL | cellular response to DNA damage stimulus | 43 | 3.55 | 8.26E-09 |
| GOTERM_BP_ALL | negative regulation of mitotic cell cycle phase transition | 20 | 8.65 | 1.09E-08 |
| GOTERM_BP_ALL | meiotic nuclear division | 21 | 7.77 | 1.97E-08 |
| GOTERM_BP_ALL | G2/M transition of mitotic cell cycle | 21 | 7.65 | 2.64E-08 |
| GOTERM_BP_ALL | negative regulation of cell cycle phase transition | 20 | 8.03 | 3.98E-08 |
| GOTERM_BP_ALL | meiotic cell cycle process | 21 | 7.40 | 4.64E-08 |
| GOTERM_BP_ALL | spindle organization | 17 | 10.30 | 4.65E-08 |
| GOTERM_CC_ALL | nuclear chromosome | 34 | 4.17 | 1.33E-08 |
| GOTERM_BP_ALL | regulation of chromosome organization | 25 | 5.78 | 5.96E-08 |
| GOTERM_BP_ALL | cell proliferation | 67 | 2.40 | 6.63E-08 |
| GOTERM_MF_ALL | tubulin binding | 25 | 5.70 | 2.77E-07 |
| GOTERM_BP_ALL | cytoskeleton-dependent cytokinesis | 12 | 19.50 | 8.18E-08 |
| GOTERM_CC_ALL | supramolecular fiber | 38 | 3.67 | 2.26E-08 |
| GOTERM_CC_ALL | polymeric cytoskeletal fiber | 38 | 3.67 | 2.26E-08 |
| GOTERM_BP_ALL | cell cycle G1/S phase transition | 23 | 6.28 | 9.20E-08 |
| GOTERM_BP_ALL | metaphase/anaphase transition of mitotic cell cycle | 12 | 19.04 | 1.06E-07 |
| GOTERM_BP_ALL | establishment of chromosome localization | 14 | 13.52 | 1.36E-07 |
| GOTERM_BP_ALL | DNA integrity checkpoint | 19 | 8.01 | 1.36E-07 |
| GOTERM_BP_ALL | chromosome localization | 14 | 13.33 | 1.62E-07 |
| GOTERM_BP_ALL | mitotic cytokinesis | 11 | 22.21 | 1.67E-07 |
