# Supplementary material for: Deep learning from HE slides predicts the clinical benefit from adjuvant chemotherapy in hormone receptor-positive breast cancer patients
Source: Sci Rep. 2021 Aug 30;11:17363. doi: 10.1038/s41598-021-96855-x (PMC8405682; doi:10.1038/s41598-021-96855-x)
Supplement: Supplementary file 2 — Supplementary Information 2. [file 41598_2021_96855_MOESM2_ESM.docx]

[Original Study]

**Deep learning from HE slides to predict the clinical benefit from adjuvant chemotherapy in hormone receptor-positive breast cancer**

**(Running Title**: Lunit-SCOPE predicts recurrence score)

Soo Youn Cho^1^, Jeong Hoon Lee^2^, Jai Min Ryu^3^, Jeong Eon Lee^3^, Eun Yoon Cho^1*^, Chang Ho Ahn^2^, Kyunghyun Paeng^2^, Sarah Lee^2^, Inwan Yoo^2^, Chan-Young Ock^2^ and Sang Yong Song^1,4,*^

^1^Department of Pathology and Translational Genomics, Samsung Medical Center, Sungkyunkwan University School of Medicine, Seoul, Korea,

^2^Lunit Inc., Seoul, Korea,

^3^Division of Breast Surgery, Department of Surgery, Samsung Medical Center, Sungkyunkwan University School of Medicine, Seoul, Korea

^4^Medical Ai Research Center, Research Institute of Future Medicine, Samsung Medical Center, Seoul, Korea

**Address correspondence to:**

**Eun Yoon Cho, M.D.** Department of Pathology and Translational Genomics, Samsung Medical Center, Sungkyunkwan University School of Medicine, 81 Irwon-ro, Gangnam-gu, Seoul, 06351, Republic of Korea

Phone: +82-2-3410-2796; Fax: +82-2-3410-0025; E-mail: eunyoon.cho@samsung.com

**Sang Yong Song, M.D.** Department of Pathology and Translational Genomics, Samsung Medical Center, Sungkyunkwan University School of Medicine, Seoul, Korea,

Phone: +82-10-9933-2803; Fax: +82-2-3410-0025; E-mail: yodasong@gmail.com

Number of tables/figures: 6

Word count: 2,843

# **SUPPLEMENTARY METHODS**

## **Data Annotation**

Manual annotations consist of 5 tissue types (cancer epithelium, cancer stroma, necrosis, and fat and normal regions), 5 structure types (tubule formation, ductal carcinoma in situ, nerve, blood vessel, and lymphatic vessel), and 8 cell types (lymph plasma cell, fibroblast, macrophage, grade 1 tumor cell, grade 2 tumor cell, grade 3 tumor cell, necrotic tumor cell, and endothelial cell). The tissue and structure types were annotated with a fine-grained pixel-level segment, and the cell type was annotated to denote the cell nucleus. We constructed 3,017 regions of 4096✕4096 pixels for tissue detection, 2,695 regions of 6144✕6144 pixels for structure detection, and 5,267 regions and 5,244 contours of 768✕768 pixels for cell detection. All of these regions were sampled at a magnification of 40✕. We split 90% of those regions for training and 10% for testing. The split was based on the whole-slide image level, not on the regional level to prevent the overlapping of slides between two sets.

## **Development of deep learning model**

For feature detection in the HE slides, we trained convolutional neural networks (CNNs), which consist of multiple convolution filters and pooling layers. Our model architecture is based on the recent state-of-the-art structure, namely EfficientNet-B3 for the encoder architecture and the DeepLab V3+ for the decoder architecture to predict pixel-level fine-grained segmentation result [1, 2]. We fully trained the entire network starting from the ImageNet (www.image-net.org) pre-trained model parameters. For the tissue and structure type training, we converted the regions to the 10✕ magnification to enlarge the field of view. For the cell type training, we converted the regions to the 20✕ magnification to capture the fine details. For the tissue and structure type, loss function was defined as the sum of the weighted cross entropy loss and the dice loss. For cell detection, the weighted cross entropy loss was used to segment the cell centroid. Diverse color augmentation and geometric augmentation were applied, such as random rotation, random crop, random scale, random brightness, random contrast, random hue, random saturation, random noise, random blur, random affine transform, and random elastic transform. We used the Adam optimization method with a fixed learning rate of 0.001. The training was done over 300 epochs for each type. We post-processed the structure segmentation results by finding the connected parameters to detect and count each structure type. The cell segmentation results were post-processed by finding local maximum peaks to detect the cell nuclei centroid. Those post-processes used the scikit-image library (www.scikit-image.org). All the convolutional neural network codes were implemented using the PyTorch library (www.pytorch.org) and were run on a desktop workstation with Nvidia Titan X GPUs. The performance for those models was evaluated using the validation set with accuracy, intersection over union (IoU), and mean average precision (mAP) with distance. When testing the whole slide, the patches were tiled into 50% overlapping 4096✕4096 pixel regions at a 40✕ magnification. The background regions were excluded by using the organ region detection model trained on the same cohort training set. Each tiled region was feed-forwarded into the trained neural networks to predict the tissue, structure, and cell types. For each slide, we extracted the feature statistics from the tissue, structure, and cell type results, such as the number of classes and the area of the classes.

## **Performance evaluation metrics**

We reported the pixel accuracy metrics from the common semantic segmentation evaluation for tissue panel and structure panel. The pixel accuracy indicates the ratio of the pixels which are correctly predicted. Let *n*_ij_ be the number of pixels of class *i* predicted to belong to class *j*. We computed te pixel accuracy for each class *i* as *n*_ii_/∑_j_*n*_ij_. For cell panel, we reported the mean average precision (mAP) metrics from the common object detection evaluation used in PASCAL VOC [3]. Average precision computed the average precision value for recall value over 0 to 1 in the precision-recall curve. PASCAL VOC metric used bounding box annotation, which cannot be used in dot annotation. We changed from the box overlap criterion to the distance criterion between the dot Ground-truth and predicted location. The distance threshold was set to 30 pixels at a 40✕ magnification. Multiple detections of the same nuclei were considered false detections. The precision-recall and the average precision were calculated.

**REFERENCES**

1. Tan M, Le Q V (2019) EfficientNet: Rethinking Model Scaling for Convolutional Neural Networks. arXiv Prepr arXiv190511946

2. Chen L-C, Collins M, Zhu Y, et al (2018) Searching for efficient multi-scale architectures for dense image prediction. In: Advances in Neural Information Processing Systems. pp 8699–8710

3. Everingham M, Eslami SMA, Van Gool L, et al (2015) The pascal visual object classes challenge: A retrospective. Int J Comput Vis 111:98–136
